# A case of irAE gastritis caused by cemiplimab administration

**DOI:** 10.20407/fmj.2025-022

**Published:** 2025-11-05

**Authors:** Kurumi Isomura, Kyohei Takada, Ryoko Ichikawa, Chiaki Oshima, Yutaro Sato, Akiko Ohwaki, Mayuko Ito, Sayaka Otani, Yusuke Shimizu, Haruki Nishizawa

**Affiliations:** Department of Obstetrics and Gynecology, Fujita Health University, School of Medicine, Toyoake, Aichi, Japan

**Keywords:** Gastritis, Upper gastrointestinal endoscopy, Immune-related adverse event, Cemiplimab

## Abstract

**Introduction:**

While various immune-related adverse events (irAEs) have been associated with the use of immune checkpoint inhibitors (ICIs), gastritis as an irAE remains a rarely documented condition. We report a case of immune-related gastritis following cemiplimab administration, accompanied by a review of the relevant literature.

**Case:**

A 75-year-old woman, gravida 4, para 2, underwent six cycles of cemiplimab as second-line treatment for FIGO stage IIB squamous cell carcinoma of the cervix. Cemiplimab was discontinued 140 days after the initial dose due to the onset of anorexia, nausea, and vomiting. However, her symptoms did not improve during the subsequent follow-up. Upper gastrointestinal endoscopy revealed erythema and edematous changes of the mucosa, predominantly in the pyloric region, along with friable, easily bleeding mucosa and white exudate. Histopathological examination of the biopsied tissue showed dense intraepithelial infiltration of CD8-positive lymphocytes. These findings were consistent with irAE gastritis. As fasting alone failed to improve the symptoms, prednisolone (PSL) was initiated, resulting in amelioration of both symptoms and endoscopic findings.

**Conclusion:**

Cemiplimab-induced immune-related gastritis can be diagnosed based on its characteristic endoscopic and histopathological features, similar to irAE gastritis caused by other immune checkpoint inhibitors.

## Introduction

Immune-related adverse events (irAEs) can affect various organs, with interstitial pneumonia, dermatologic disorders, and thyroid dysfunction being common manifestations. However, reports of gastritis as an irAE remain limited. As of April 2025, cases of irAE gastritis associated with nivolumab, pembrolizumab, and ipilimumab have been documented, but no reports of cemiplimab-induced irAE gastritis were identified in a PubMed search. Herein, we report a case of irAE gastritis that developed following cemiplimab administration, along with a review of the relevant literature.

## Case

**Figure 1:**
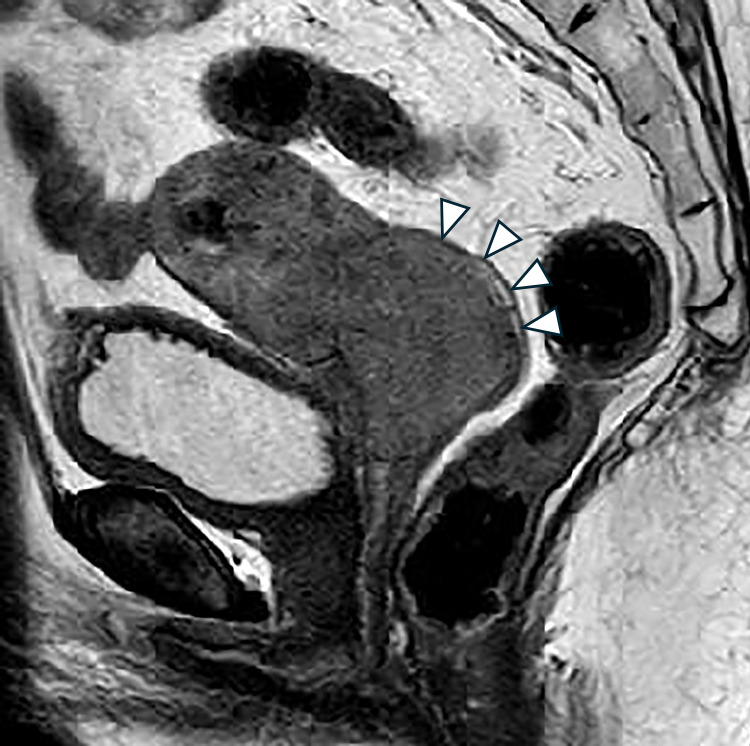
Non-contrast sagittal T2-weighted MRI of the pelvic region showing a cervical mass (arrowhead).

**Figure 2:**
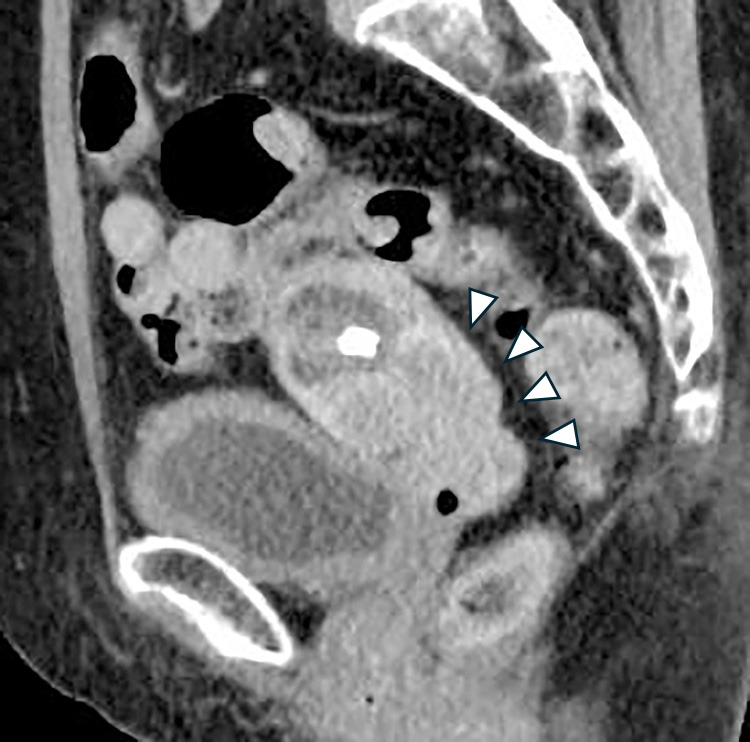
Non-contrast sagittal CT of the pelvic region showing a cervical mass (arrowhead).

**Figure 3:**
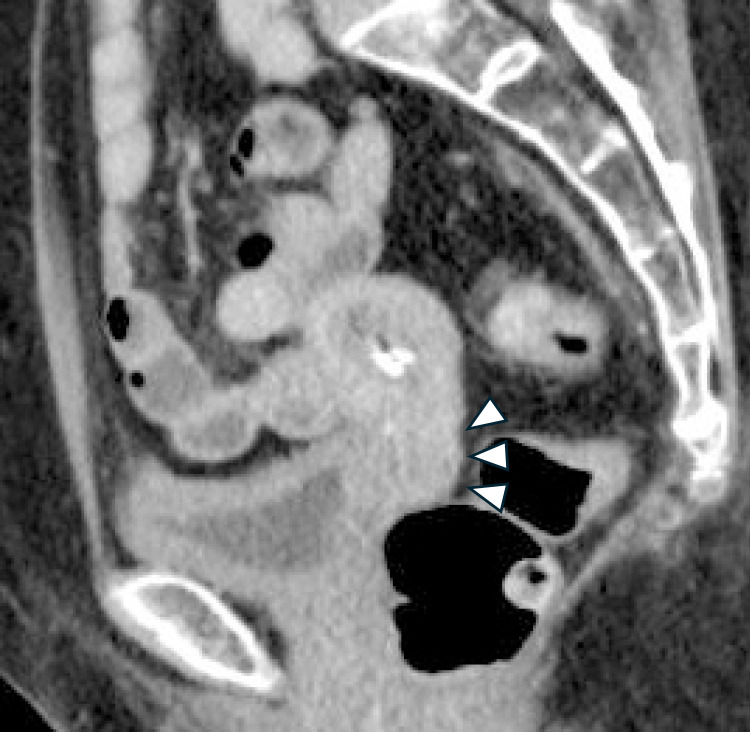
Non-contrast sagittal CT of the pelvic region showing a reduced cervical mass after cemiplimab treatment (arrowhead).

**Figure 4:**
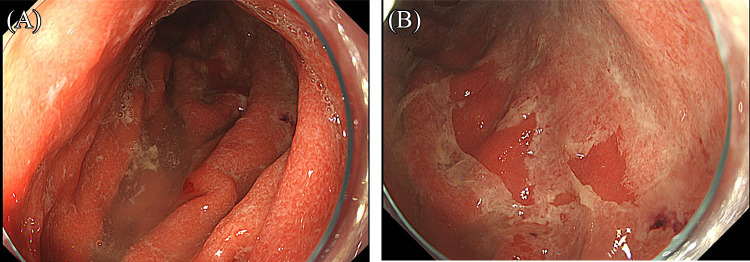
(A) Upper gastrointestinal endoscopic findings showing erythema and edematous changes of the mucosa, predominantly in the pyloric region. (B) Upper gastrointestinal endoscopic findings showing fragile, easily bleeding mucosa with white exudate.

**Figure 5:**
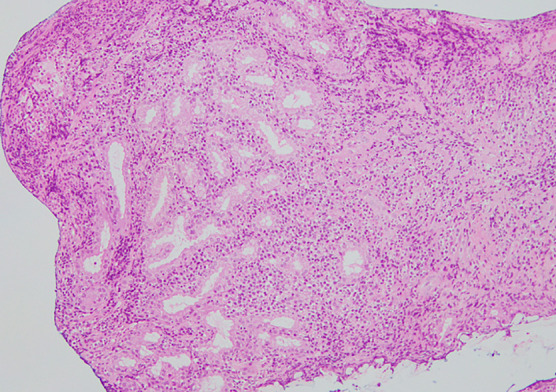
Pathologic findings from a gastric biopsy (HE staining) showing interstitial infiltration of inflammatory cells, including numerous lymphocytes, plasma cells, and neutrophils.

**Figure 6:**
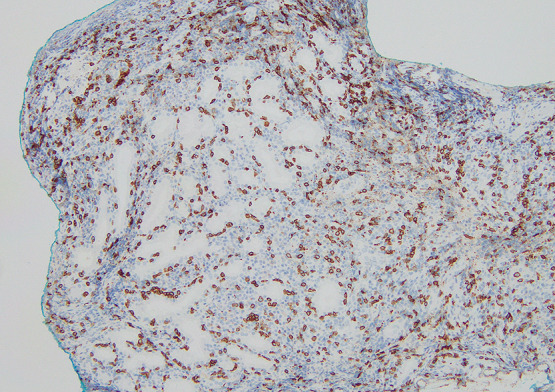
Pathologic findings from a gastric biopsy (immunohistochemistry) showing marked intraepithelial infiltration of CD8-positive lymphocytes.

**Figure 7:**
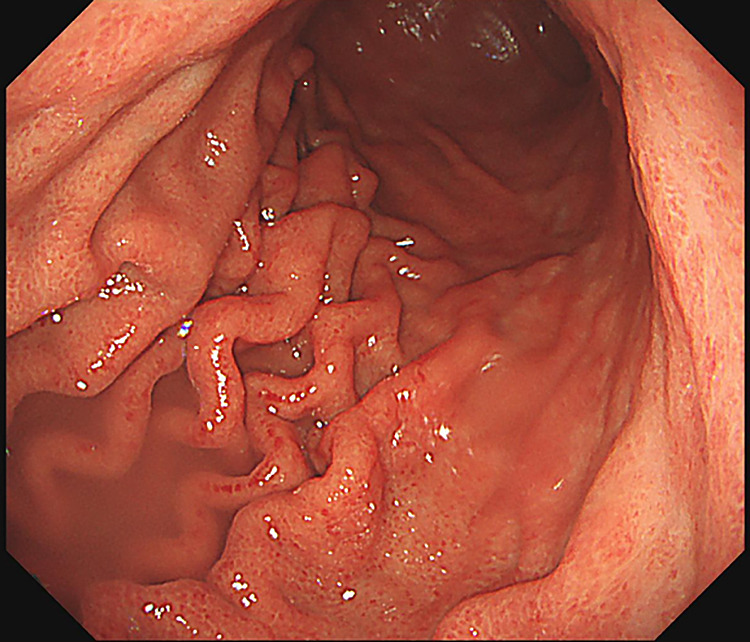
Upper gastrointestinal endoscopic findings showing improvement in redness and edematous changes around the pylorus—previously the most inflamed area—following prednisolone (PSL) administration.

A 75-year-old woman (gravida 4, para 2) was suspected of having squamous cell carcinoma (SCC) based on cervical cancer screening and subsequently underwent further examination. MRI revealed a 50-mm mass in the uterine cervix with parametrial invasion (Figure 1). The clinical stage was diagnosed as FIGO stage IIB, and the TNM classification was cT2bN0M0. Concurrent chemoradiotherapy (CCRT) was initiated as first-line therapy but was discontinued at the patient’s request due to a gastric ulcer, which occurred as an adverse event after two cycles of cisplatin (40 mg/m^2^) and whole pelvic irradiation (12.6 Gy/7 fractions). The patient later expressed a desire to resume treatment for the residual cervical tumor. She received six cycles of combination chemotherapy with paclitaxel and carboplatin (TC regimen). Computed tomography (CT) subsequently showed a partial response (PR), and maintenance therapy with bevacizumab was administered for 15 cycles but discontinued due to Grade 2 proteinuria. Follow-up CT revealed progression, with the cervical tumor increasing to 37 mm in diameter (Figure 2) and the right obturator lymph node enlarging to 20 mm, leading to a diagnosis of progressive disease (PD). The patient was then treated with six cycles of cemiplimab at a dose of 350 mg. After six cycles, the cervical mass was reduced to 10 mm, and the right obturator lymph node decreased to 7 mm (Figure 3). On the 140th day following initial cemiplimab administration, the patient developed anorexia and vomiting. Although a proton pump inhibitor (PPI) was prescribed, her symptoms progressively worsened. Upper gastrointestinal (UGI) endoscopy, performed 22 days after symptom onset, revealed mucosal erythema and edematous changes, predominantly in the pyloric region (Figure 4A, as well as friable, easily bleeding mucosa with white exudate (Figure 4B). Histopathological examination of a gastric biopsy specimen demonstrated inflammatory infiltration of the lamina propria by numerous lymphocytes, plasma cells, and neutrophils, as visualized by hematoxylin and eosin (HE) staining (Figure 5). Immunohistochemical analysis showed dense intraepithelial infiltration of CD8-positive lymphocytes (Figure 6). These findings led to a diagnosis of irAE gastritis. Fasting combined with PPI therapy for 10 days failed to alleviate symptoms. Therefore, prednisolone (1 mg/kg/day) was initiated approximately one month after symptom onset. The symptoms improved following the initiation of prednisolone (PSL), and a follow-up upper gastrointestinal endoscopy performed approximately 3 weeks later showed resolution of the erythema and edematous changes around the pylorus, where inflammation had been most pronounced (Figure 7). In this case, the patient was diagnosed with Grade 3 irAE gastritis. As the condition did not resolve to Grade 1 or lower despite treatment interruption exceeding 12 weeks, cemiplimab was permanently discontinued.

## Discussion

Cemiplimab can cause a variety of immune-related adverse events (irAEs); however, upper gastrointestinal involvement is rare, occurring in only 1.1%–1.4% of ICI-treated patients.^1^ In cases of irAE gastritis, nausea and vomiting occur in approximately 80% of patients, with other symptoms including anorexia and abdominal pain.^2^ The mean interval from ICI initiation to the onset of irAE gastritis has been reported as 132 days.^3^ In the present case, gastritis developed 140 days after initiation, consistent with previous reports. As the symptoms of irAE gastritis are nonspecific, diagnosis should be based on clinical course, endoscopic findings, and histopathological examination.^4^ Differential diagnoses include Helicobacter pylori gastritis, cytomegalovirus or Epstein–Barr virus gastritis, and inflammatory bowel disease.^2^

A diagnosis of irAE gastritis can be established based on characteristic endoscopic and histopathological findings. Regarding endoscopic features, Farha et al.^2^ reported erythema, edema, and mucosa with a tendency to bleed easily, while Sugiyama et al.^4^ described reticulated erosions or ulcers in the pyloric region, erythematous and edematous mucosa with excessive white purulent discharge throughout the stomach, and severely erosive mucosa. As for histopathological findings, Farha et al.^2^ observed intraepithelial lymphocytic infiltration, apoptosis, and mild to moderate inflammation of the lamina propria. Furthermore, Irshaid et al.^5^ reported an increased number of CD8-positive intraepithelial lymphocytes, reduced inflammation in the lamina propria, fewer plasma cells and CD20-positive B cells, fewer lymphoid aggregates, and a decreased CD4:CD8 ratio in both the lamina propria and epithelial layers. Histopathological examination plays a critical role in the differential diagnosis. *H. pylori* gastritis is typically characterized by neutrophilic infiltration or a mixed infiltrate of neutrophils and lymphocytes. CMV or EBV gastritis is marked by lymphoplasmacytic infiltration of the lamina propria with involvement of glandular structures, along with atypical lymphocytes possessing prominent nucleoli. Inflammatory bowel disease may be associated with characteristic epithelioid granulomas.^2,6^ CMV gastritis must be excluded, as CMV reactivation is a known risk in patients with malignancies receiving ICI therapy.^7^

In the present case, endoscopic findings revealed erythema and edematous changes of the mucosa, predominantly in the pyloric region, along with easily bleeding, fragile mucosa and white exudate. Histopathological examination with hematoxylin and eosin (HE) staining demonstrated interstitial infiltration of inflammatory cells, including numerous lymphocytes, plasma cells, and neutrophils Apoptosis-like changes in glandular epithelial cells were also observed, although definitive interpretation was challenging due to the severity of the inflammation. Immunohistochemical analysis revealed marked intraepithelial infiltration of CD8-positive lymphocytes. These findings are consistent with those previously reported and suggest that cemiplimab-induced irAE gastritis exhibits pathological and endoscopic features similar to those seen with nivolumab-, pembrolizumab-, and ipilimumab-induced irAE gastritis. Although cemiplimab was discontinued in the present case due to Grade 3 irAE gastritis, resumption may be considered if the condition improves to Grade 1 within a 12-week interruption period. However, as irAE gastritis carries a risk of recurrence, close and continuous follow-up is essential. For future treatment, nogitecan and tisotumab vedotin^8^ may be considered as potential therapeutic options.

## Conclusion

Cemiplimab-induced irAE gastritis can be diagnosed based on its characteristic endoscopic and histopathological findings.

## Supplementary Material

PDF-Japanese
