# Supplementary material for: A case of irAE gastritis caused by cemiplimab administration
Source: Fujita Med J. 2025 Nov 5;12(1):84–7. doi: 10.20407/fmj.2025-022 (PMC12865286; doi:10.20407/fmj.2025-022)
Supplement: Supplementary file 1 — PDF-Japanese [file fmj-12-084-s001.pdf]

Case report

Cemiplimab 投与で発症した irAE 胃炎の 1 例

ランニングタイトル

特徴的な内視鏡所見と病理学的所見により診断された症例

Kurumi Isomura, M.D., Kyohei Takada, M.D., Ryoko Ichikawa, M.D., Chiaki Oshima,M.D., Yutaro Sato, M.D., Akiko Owaki, M.D., Ph.D., Mayuko Ito, M.D., Ph.D., Sayaka Otani,M.D.,Ph.D., Yusuke Shimizu, M.D., Ph.D., Haruki Nishizawa, M.D., Ph.D.

Department of Obstetrics and Gynecology, School of Medicine, Fujita Health University

Corresponding author: Ryoko Ichikawa, M.D.

Fujita Health University Hospital

1-98 Dengakugakubo, Kutsukake-cho, Toyoake, Aichi 470-1192, JAPAN

TEL: +81-562-93-2111

kurumi.isomura@fujita-hu.ac.jp

概要

【諸言】免疫チェックポイント阻害薬(ICI)の使用により様々な免疫関連有害事象(irAE)が発症しうることが知られているが、irAE としての胃炎の報告は少ない。今回、Cemiplimab 投与後に発症した irAE 胃炎を経験したので、文献的考察を含めて報告する。

【症例】75 歳、4 妊 2 産。FIGO stage IIB の子宮頸部扁平上皮癌の second line の治療として Cemiplimab６サイクルを施行した。初回投与から 140 日後に食思不振や悪心嘔吐を認めたため、休薬し経過観察したが改善しなかった。上部消化管内視鏡検査を施行したところ、幽門部中心の粘膜の紅斑と浮腫状変化、易出血性で脆弱な粘膜と白色滲出液が見られた。生検した組織は多数の CD8 陽性リンパ球が上皮内浸潤していた。これらは irAE 胃炎として典型的な所見であった。絶食管理としたが改善しないため Prednisolone（PSL）を投与し症状および内視鏡所見の改善を認めた。

【結語】Cemiplimab による irAE 胃炎は他の ICI 同様特徴的な内視鏡所見および病理学的所見により診断可能である。

Key word: 胃炎、上部消化管内視鏡、免疫関連有害事象、セミプリマブ

【諸言】免疫関連有害事象（immune-related Adverse Events : irAE）は全身の臓器に出現し、代表的なものとして間質性肺炎、皮膚障害、甲状腺機能障害などがあるが胃炎の報告は少ない。2025 年 4 月までに Nivolumab、Pembrolizumab、Ipilimumab による irAE 胃炎の報告はあるが、Cemiplimab による報告は pubmed 検索ではない。今回、Cemiplimab 投与後に発症した irAE 胃炎を経験したので文献的考察を含めて報告する。

【症例】現病歴：75 歳。4 妊 2 産。子宮頸癌検診で SCC と判定され精査したところ、MRI で子宮頸部に最大径 50mm の子宮傍組織浸潤のある腫瘤（figure1）を認めた。進行期は FIGO stage IIB、TNM 分類 cT2bN0M0 と診断した。初回治療として同時化学放射線療法（concurrent chemoradiotherapy：CCRT）を開始したが、全骨盤照射 12.6Gy/7Fr および Cisplatin40mg/m^2 2 サイクル施行後、胃潰瘍の有害事象を認め患者希望で中止した。その後頸部残存腫瘍に対して治療再開希望あり、Paclitaxel Carboplatin 併用療法（TC 療法）を開始し計６サイクル施行した。その後 CT で部分奏効（Partial Response：PR）であったため Bevacizumab 維持療法を 15 サイクル施行したが、Grade2 の蛋白尿があり中止し、CT で子宮頸部腫瘤は最大径 37mm に（figure2）、右閉鎖リンパ節は 20mm に増大し進行（progressive disease:PD）あったため Cemiplimab 350mg を 6 サイクル施行した。Cemiplimab 6 サイクル施行後、奏効し子宮頸部腫瘤は最大径 10mm に、右閉鎖リンパ節は 7mm(figure3)に縮小した。Cemiplimab 初回投与 140 日目から食思不振と嘔吐が出現し、プロトンポンプ阻害薬（proton pump inhibitor:PPI）内服していたが、症状の増悪あり症状発現から 22 日後に上部消化管内視鏡検査を施行した。上部消化管内視鏡所見では、幽門部中心に粘膜の紅斑と浮腫状変化がみられ(figure4-1)、また易出血性で脆弱な粘膜と白色滲出液が見られた(figure4-2)。胃の生検組織の病理学的所見においては、ヘマトキシリン・エオジン（Hematoxylin Eosin：HE）染色で間質に多数のリンパ球、形質細胞、好中球が混在する炎症性細胞浸潤が見られた(figure5)。免疫組織化学では CD8 陽性リンパ球が上皮内に多数浸潤していた（figure6）。以上の内視鏡、病理組学的所見より irAE 胃炎と診断した。絶食と PPI 投与を 10 日間施行したが症状改善なく、発症後約 1 ヶ月で Prednisolone（PSL）を 1mg/kg/day で開始した。PSL 開始後から症状は改善し、PSL 開始後から約 3 週間の上部消化管内視鏡所見においても特に炎症の強かった幽門付近の発赤や浮腫状変化は改善した（figure7）。本症例は Grade3

の irAE 胃炎であり、12 週間を超える休薬後も Grade1 以下まで回復しなかったため Cemiplimab の投与は中止となった。

【考察】Cemiplimab は様々な irAE を引き起こしうるが、上部消化管の irAE は ICI 投与症例の 1.1-1.4%と稀である[1)]。irAE 胃炎は 8 割に悪心・嘔吐を認め、その他食思不振や腹痛が生じ[2)]、ICI 投与開始から発症までの期間は平均 132 日と報告されている[3)]。本症例は ICI 投与開始から約 140 日で発現しており既報と同様の経過であった。irAE 胃炎の症状は非特異的であり、診断は患者の臨床経過、内視鏡所見および病理組織学的所見に基づいて行うべきである[4)]。鑑別疾患には Helicobacter pylori 感染性胃炎、Cytomegalovirus または Epstein Barr virus 胃炎、炎症性腸疾患があがるが[2)]、内視鏡所見および病理組織学的所見により irAE 胃炎は診断可能である。irAE 胃炎の内視鏡所見として、Farha ら[2)]は紅斑、浮腫、易出血性であると報告しており、杉山ら[4)]は幽門部の網目状びらんまたは潰瘍、胃全体に過剰な白色膿性分泌物を伴う紅斑性・浮腫性粘膜、極度のびらん性粘膜であると報告している。病理組織学的所見としては Farha ら[2)]はリンパ球の上皮内浸潤、アポトーシス、粘膜固有層の炎症が軽度から中等度であると報告している。また、Irshaid ら[5)]は CD8 陽性上皮内リンパ球の数が多く、粘膜固有層の炎症が少なく、形質細胞および CD20 陽性 B 細胞の数が少なく、リンパ球凝集巣が少なく、粘膜固有層および上皮層の両方で CD4:CD8 比が減少していると報告している。病理学的所見で鑑別が可能で、Helicobacter pylori 感染性胃炎では好中球性浸潤または好中球とリンパ球の混合性浸潤を伴うことが多く、Cytomegalovirus や Epstein Barr virus 胃炎では一部の腺浸潤を伴う固有層にリンパ形質細胞浸潤を伴い、顕著な核小体を有する異型リンパ球の存在し、炎症性腸疾患では特徴的な上皮様肉芽腫が挙げられる[2),6)]。Cytomegalovirus は ICI 治療を受けている悪性腫瘍患者において再活性化するリスクがあるため除外が必要である[7)]。本症例は内視鏡所見として、幽門部中心に粘膜の紅斑と浮腫状変化、易出血性で脆弱な粘膜と白色滲出液が見られ、病理学的所見として HE 染色では間質に多数のリンパ球、形質細胞、好中球が混在する炎症性細胞浸潤が見られた。また、炎症が強く確定は困難であるが腺上皮細胞のアポトーシス様の像が見られた。免疫組織化学では多数の CD8 陽性リンパ球の上皮内浸潤が見られた。これらの所見は既報と同様であり、Cemiplimab においても Nivolumab、Pembrelizumab、Ipilimumab による irAE 胃炎と同様の所見となることがわかった。本

症例は irAE により Cemiplimab は投与中止となったが、Grade3 の irAE 胃炎は 12 週以内の休薬で Grade1 まで回復する場合は再開可能である。ただし、irAE 胃炎は再燃リスクがあるため継続的な経過観察が必要である。今後は、Nogitecan や Tisotumab vedotin[8)]が次の治療の選択肢になると考える。

【結語】Cemiplimab による irAE 胃炎は特徴的な内視鏡所見と病理学的所見により診断可能である。

【利益相反】この論文に関連して開示すべき利益相反状態はありません。

【謝辞】本症例の報告にご協力いただいた患者様に心より感謝申し上げます。また、本症例の執筆にあたりご指導を賜りました先生方に深謝いたします。

【文献】

1. Sakai M, Haga Y, Kambe M, Nishimura K, Shingyouchi A, Miyamura T, Ito K,Abe A,Kaneda S,Tada M,Saito M,Sugiura N. A case of immune-related adverse effect diffuse gastritis induced by nivolumab. Progress of Digestive Endoscopy 2021 Jun 25;98(1):91–2. (in Japanese)
2. Farha N, Faisal MS, Allende DS, Sleiman J, Shah R, Farha N, Funchain P, Philpott J. Characteristics of Immune Checkpoint Inhibitor-Associated Gastritis: Report from a Major Tertiary Care Center. The Oncologist. 2023 Aug 3;28(8):706–13.
3. Collins M, Michot JM, Danlos FX, et al. Inflammatory gastrointestinal diseases associated with PD-1 blockade antibodies. Ann Oncol. 2017 Nov;28(11):2860–5.
4. Sugiyama Y, Tanabe H, Matsuya T, et al. Severe immune checkpoint inhibitor-associated gastritis: A case series and literature review. Endosc Int Open. 2022 Jul;10(07):E982–9.
5. Irshaid L, Robert ME, Zhang X. Immune Checkpoint Inhibitor–Induced Upper Gastrointestinal Tract Inflammation Shows Morphologic Similarities to, but Is Immunologically Distinct From, *Helicobacter pylori* Gastritis and Celiac Disease. Arch Pathol Lab Med. 2021 Feb 1;145(2):191–200.
6. Polydorides AD. Erratum to “Pathology and differential diagnosis of chronic, noninfectious gastritis” [Seminars in Diagnostic Pathology 31 (2014) 114–123]. Semin Diagn Pathol. 2014 Jul;31(4):314–7.
7. Egoryan G, Zimmet A, Yu M, Pozdol J, Subramanian A, Reddy S, Nelson J. A Novel Intersection: Cytomegalovirus Gastritis Following Cemiplimab and Talimogene Laherparepvec in a Patient With Advanced Cutaneous Squamous Cell Carcinoma. Clin

Case Rep. 2024 Dec;12(12):e9632.

8. Coleman RL, Lorusso D, Gennigens C, et al. Efficacy and safety of tisotumab vedotin in previously treated recurrent or metastatic cervical cancer (innovaTV 204/GOG-3023/ENGOT-cx6): a multicentre, open-label, single-arm, phase 2 study. Lancet Oncol. 2021 May;22(5):609–19.

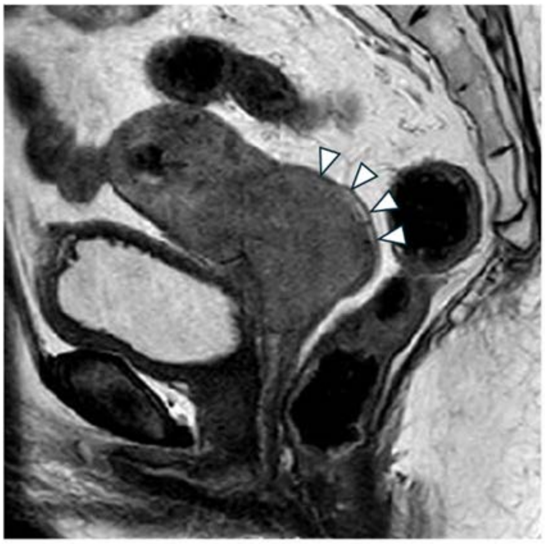

Figure.1
骨盤部単純MRI T2強調像矢状断：子宮頸部腫瘤（矢頭）

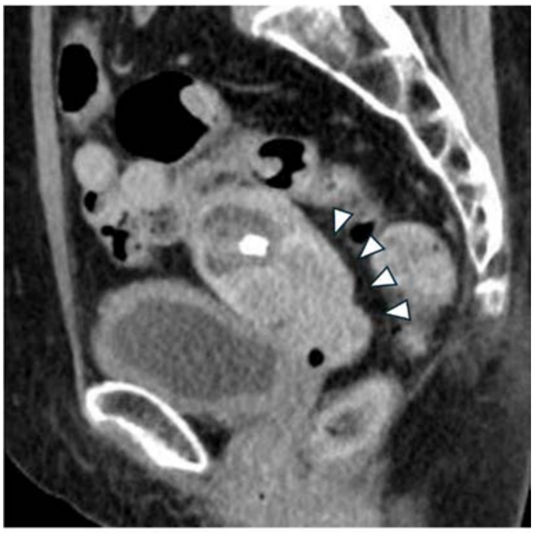

Figure.2
骨盤部単純CT　矢状断：子宮頸部腫瘤（矢頭）

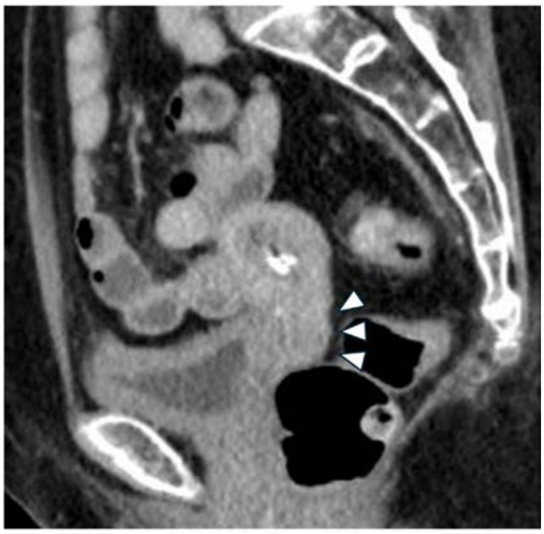

Figure.3
骨盤部単純CT　矢状断：子宮頸部腫瘤（矢頭）

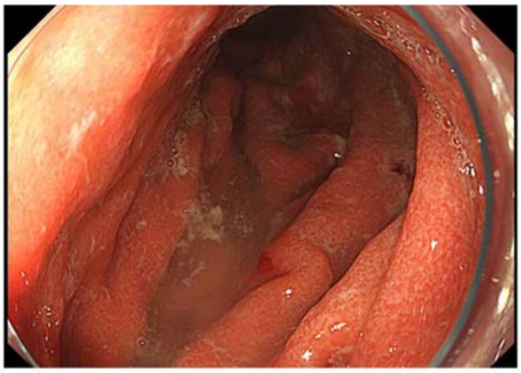

Figure.4-1
上部消化管内視鏡所見：幽門部中心に粘膜の紅斑と浮腫状変化が見られる。

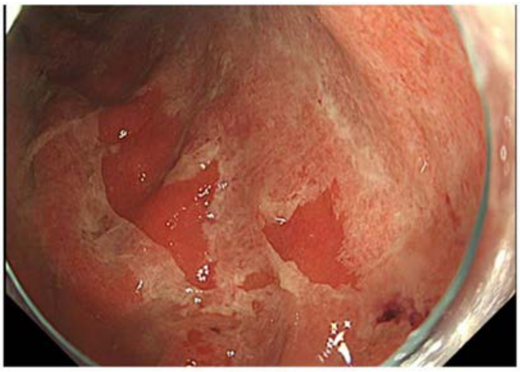

Figure.4-2
上部消化管内視鏡所見：易出血性の脆弱な粘膜と白色滲出液が見られる。

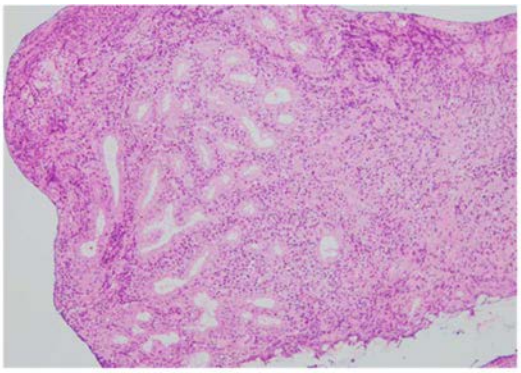

Figure.5
胃生検組織の病理学的所見（HE染色）：間質に多数のリンパ球、形質細胞、好中球が混在する炎症性細胞の浸潤が見られる。

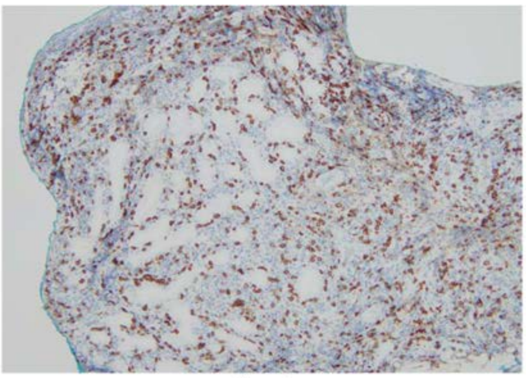

Figure.6
胃生検組織の病理学的所見（免疫組織化学）：多数のCD8陽性リンパ球が上皮内に浸潤している。

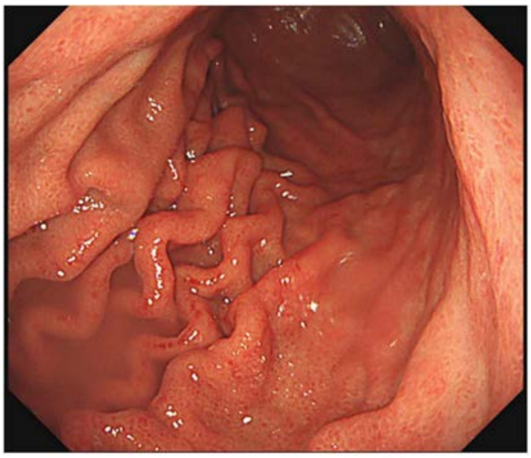

Figure.7
上部消化管内視鏡所見 ：PSLによって特に炎症の強かった幽門付近の発赤や浮腫状変化が改善した。
